# Supplementary material for: Investigating Primary Cilia during Peripheral Nervous System Formation
Source: Int J Mol Sci. 2021 Mar 20;22(6):3176. doi: 10.3390/ijms22063176 (PMC8003989; doi:10.3390/ijms22063176)
Supplement: Supplementary file 1 [file ijms-22-03176-s001.pdf]

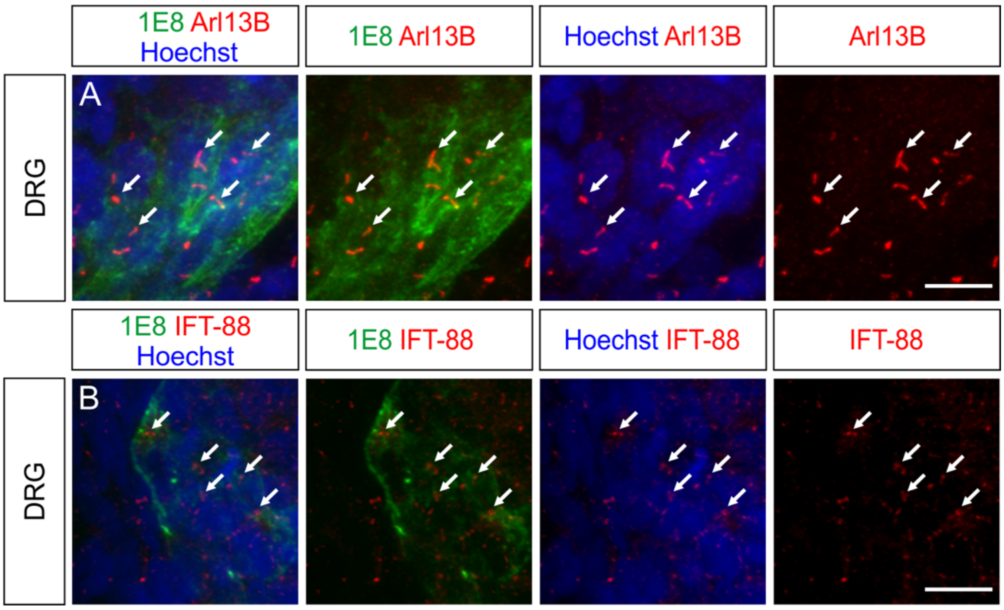

Figure S1. 1E8-positive Schwann cell precursors located in DRG are ciliated. (**A**), (**B**) Transverse sections of HH26 embryos stained for 1E8 (green), Arl13B (**A**), or IFT88 (**B**) (red) and counterstained with Hoechst (blue). This revealed that 1E8-positive Schwann cell precursors bore a primary cilium in developing the DRG (white arrows). Scale bars: 10 µm.

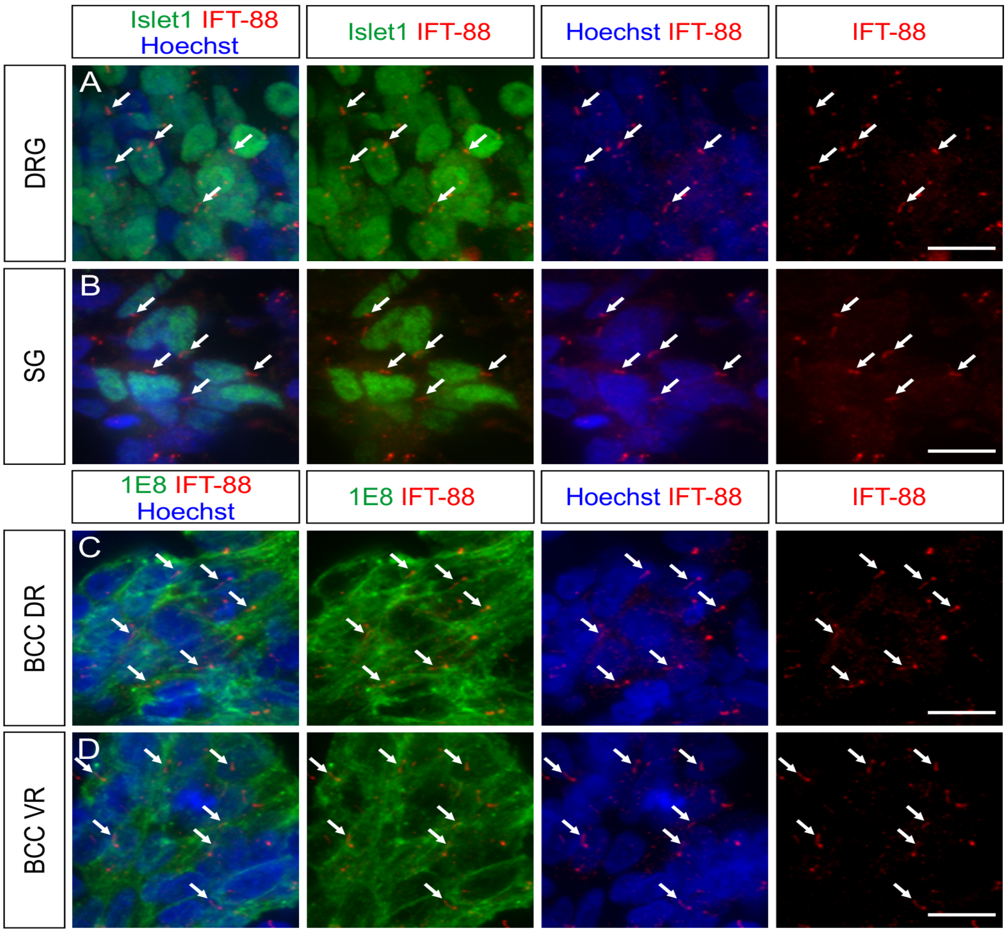

Figure S2. Confirmation of the presence of a primary cilium on DRG neurons, SG neurons, and BCC with IFT88 staining. IFT88 is a component of the intraflagellar transport complex (**A**). (**B**) Transverse sections of HH26

embryos stained for Islet-1 (green) and IFT88 (red) and counterstained with Hoechst (blue). IFT88 staining confirmed that Islet-1-positive DRG neurons (**A**) and SG neurons (**B**) bore a primary cilium (white arrows). (**C**), (**D**) Transverse sections of HH26 embryos stained for 1E8 (green) and IFT88 (red) and counterstained with Hoechst (blue). IFT88 staining confirmed that 1E8-positive BCCs bore a primary cilium (white arrows) at the dorsal (**C**) and ventral roots (**D**). d, dorsal; v, ventral; DR, dorsal roots; VR, ventral roots. Scale bars: 10 µm.

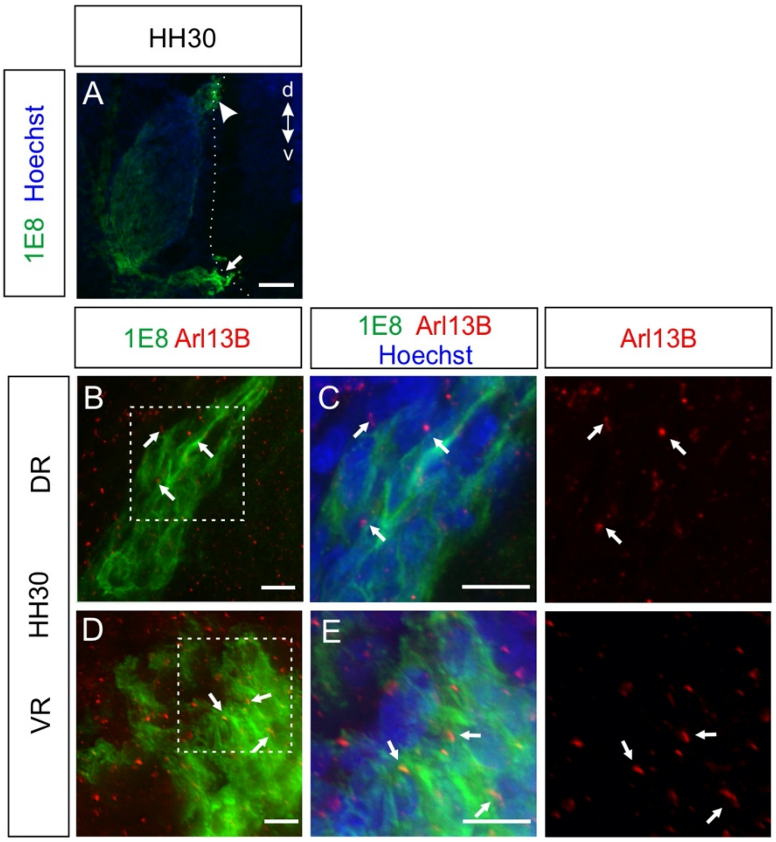

Figure S3. Boundary cap cells (BCCs) carry a primary cilium at HH30, in both dorsal and ventral roots. (**A**) 1E8-positive BCC cluster (green) localized at the dorsal root (DR, arrowhead) and the ventral root (VR, arrow). High-magnification micrographs of BCCs at DR (**B**,**C**) and VR (**D**,**E**), colabeled with Arl13B (red), revealed that these cells carried a primary cilium at HH30 (white arrows). Dashed line in A represents the boundary of the spinal cord. Squares with dashed lines represent the region of interest in the right panels. d, dorsal; v, ventral; VR, central roots; DR, dorsal roots. Scale bars: 100 (**A**) and 10 µm (**B**–**E**).

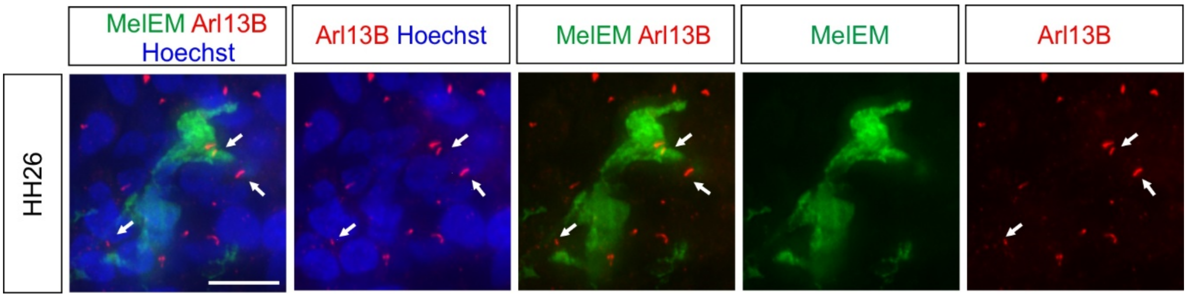

Figure S4. Melanocytes possess a primary cilium in vivo. MelEM stained melanocytes (green) and Arl13B (red) visualized primary cilia (arrows). Hoechst stained nuclei (blue). Stage: HH26. Scale bar: 10 µm.
